# ENHANCING POSITIVE AFFECT AND CAREGIVER EFFICACY (ENHANCE): INTERVENTION DEVELOPMENT FEEDBACK

**DOI:** 10.1093/geroni/igae098.2411

**Published:** 2024-12-31

**Authors:** Hannah Mason, Ana Sophia Garces Torres, E-Shien Chang, Sara Czaja

**Affiliations:** Weill Cornell Medicine, New York, New York, United States; Weill Cornell Medicine/Center on Aging and Behavioral Research, New York, New York, United States; Weill Cornell Medical College, New York, New York, United States; Weill Cornell Medicine/Center on Aging and Behavioral Research, New York, New York, United States

## Abstract

Currently about 11.5 million informal caregivers are providing care for older adults with Alzheimer’s Disease and Related Dementias (ADRD). The overburdening of these caregivers fosters an urgency for assistive programs and services. Two important determinants of caregivers’ use of available services are accessibility and relevance, both having potential to be a facilitator or barrier to service use. To address the need for accessible and efficacious caregiver interventions, the Enhancing Positive Affect and Caregiver Efficacy (ENHANCE) intervention program is being developed. ENHANCE is a multicomponent, technology-based and personalized intervention that offers self-guided opportunities and one-on-one sessions to deliver caregiving skills, access to resources, and intrapersonal support. As part of the development process, two focus groups (n=11) were conducted with ADRD caregivers to identify caregiving challenges and obtain feedback on the ENHANCE intervention prototype. This presentation will discuss findings from the focus groups. Thematic analysis revealed three common challenges among the caregivers: (1) identity loss and emotional repression, (2) communication difficulties particularly with family and healthcare providers, and (3) unreliable formal support services. Caregivers were enthusiastic about the topics included in the ENHANCE program, particularly legal/financial planning and communication. Several participants wanted to meet again, highlighting the value of open discussion among caregivers and the importance of having their voices at the table when developing caregiving services. Incorporating caregivers’ perspectives to aid the development of caregiving interventions like the ENHANCE program can improve their design, utility, and accessibility.

